# Association between HLA gene polymorphisms and mortality of COVID‐19: An in silico analysis

**DOI:** 10.1002/iid3.358

**Published:** 2020-10-13

**Authors:** Yusuke Tomita, Tokunori Ikeda, Ryo Sato, Takuro Sakagami

**Affiliations:** ^1^ Department of Respiratory Medicine, Graduate School of Medical Sciences Kumamoto University Kumamoto Japan; ^2^ Laboratory of Clinical Pharmacology and Therapeutics, Faculty of Pharmaceutical Sciences Sojo University Kumamoto Japan; ^3^ Department of Medical Information Sciences and Administration Planning Kumamoto University Hospital Kumamoto Japan; ^4^ Laboratory of Stem Cell and Neuro‐Vascular Biology, Genetics and Developmental Biology Center, National Heart, Lung, and Blood Institute National Institutes of Health Bethesda Maryland USA

**Keywords:** COVID‐19, human leukocyte antigen, pandemic, severe acute respiratory syndrome coronavirus 2, T cell

## Abstract

**Introduction:**

The emergence of SARS‐CoV‐2 has caused global public health and economic crisis. Human leukocyte antigen (HLA) is a critical component of the viral antigen presentation pathway and plays essential roles in conferring differential viral susceptibility and severity of diseases. However, the association between HLA gene polymorphisms and risk for COVID‐19 has not been fully elucidated. We hypothesized that HLA genotypes might impact on the differences in morbidity and mortality of COVID‐19 across countries.

**Methods:**

We conducted in silico analyses and examined an association of HLA gene polymorphisms with prevalence and mortality of COVID‐19 by using publicly available databases.

**Results:**

We found that a possible association between HLA‐A*02:01 and an increased risk for COVID‐19. HLA‐A*02:01 had a relatively lower capacity to present SARS‐CoV‐2 antigens compared with other frequent HLA class I molecules, HLA‐A*11:01 or HLA‐A*24:02.

**Conclusion:**

This study suggests that individuals with HLA‐A*11:01 or HLA‐A*24:02 genotypes may generate efficiently T‐cell‐mediated antiviral responses to SARS‐CoV‐2 compared with HLA‐A*02:01. The differences in HLA genotypes may potentially alter the course of the disease and its transmission.

## Graphical abstract

We conducted in silico analyses and examined an association of HLA gene polymorphisms with prevalence and mortality of COVID‐19 by using publicly available databases. We found that the association between HLA‐A*02:01 and increased risk for COVID‐19 is probably due to the lower capacity of the genotype to present SARS‐CoV‐2 antigens. Our study suggests that identifying the HLA genotype associated with the severity of COVID‐19 or susceptibility to SARS‐CoV‐2 may support future vaccination strategies to genotypically at‐risk populations.

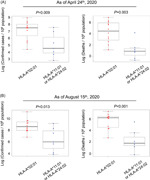

## 1 INTRODUCTION

The emergence of severe acute respiratory syndrome coronavirus 2 (SARS‐CoV‐2) and its spread have caused global public health and economic crisis.^1^, ^2^, ^3^ Many countries were being forced to introduce strict limitations to reduce the risk of contagion, and the coronavirus disease 2019 (COVID‐19) was considered pandemic since March 11, 2020.^1^, ^2^ As of August 20, 2020, there are over 22.2 million confirmed cases of COVID‐19 worldwide, with total deaths exceeding 781,000 (WHO Coronavirus Disease (COVID‐19) Dashboard; https://covid19.who.int). Given the severity of the disease, understanding host immune response and the rapid development of effective vaccines against SARS‐CoV‐2 are urgently needed.^1^

The COVID‐19 pandemic originated in China and it has quickly spread all over the world.^2^ However, there are striking differences in the morbidity and mortality of COVID‐19 among countries (WHO Coronavirus Disease (COVID‐19) Dashboard; https://covid19.who.int).^2^ For instance, Italy, Spain, and France have imposed nationwide lockdown to combat the spread of SARS‐CoV‐2, but COVID‐19 morbidity and mortality are still high. Japan is one of the first countries that recorded the first case in the earliest phase of this pandemic, but the morbidity and mortality are still low despite the Japanese government just keeps applying mild measures to mitigate the spread of the virus. A number of factors have been proposed to impact the morbidity and mortality of SARS‐CoV‐2, and these include geographical, social, and biological factors such as temperature, humidity, life expectancy, average income, social norms, Bacillus Calmette‐Guérin (BCG) vaccination policy, and genetic factors.^2^, ^4^, ^5^

In this study, we hypothesized that HLA genotypes may differentially induce the T‐cell‐mediated antiviral response to SARS‐CoV‐2, and could potentially impact the prevalence of COVID‐19 and mortality by SARS‐CoV‐2 among countries. In other words, if the most common HLA alleles in the population could provide a protective effect on COVID‐19, a significant reduction of the spread of SARS‐CoV‐2 and a reduced mortality rate may be induced, and then the differences in the frequency of HLA allele resistant to COVID‐19 lead to the country‐by‐country difference in COVID‐19 morbidity and mortality. If HLA genetic variation affects the course of COVID‐19, HLA typing could improve the assessment of viral severity in the population and could help identify individuals at higher risk from the disease. Individuals with high‐risk HLA types could be prioritized for vaccination after developing vaccines against SARS‐CoV‐2.^6^ Therefore, understanding the impact of HLA gene polymorphisms on the differences in morbidity and mortality of SARS‐CoV‐2 among countries is a critical and urgent challenge in COVID‐19 pandemic.^1^, ^7^

Human leukocyte antigen (HLA) is a critical component of the viral antigen presentation pathway and plays essential roles in conferring differential viral susceptibility and severity of diseases.^6^, ^7^, ^8^, ^9^ It is considered that the diversity of HLA molecules at the population level makes it possible to maximize the probability that at least some individuals within the general population mount an immune attack against an emerging infection and can survive.^6^, ^7^ Importantly, polymorphisms in the HLA have been shown to impact susceptibility to SARS‐CoV introduced to humans earlier in the 21st century.^6^, ^7^ In disease caused by SARS‐CoV in 2003, increased severity among individuals with HLA‐B*46:01 has been shown.^8^ In addition, studies have demonstrated a possible association between HLA gene polymorphisms and susceptibility to SARS‐CoV.^4^, ^9^ Recently, Nguyen et al.^7^ have reported that HLA‐B*46:01 as the HLA allele with the fewest predicted binding peptides for SARS‐CoV‐2 as well as SARS‐CoV. These results suggest that pairing HLA typing with COVID‐19 testing could improve assessment of the severity of the disease caused by SARS‐CoV‐2.^7^, ^9^ However, the association between HLA gene polymorphisms and risk for COVID‐19 has not been fully elucidated.^1^, ^2^, ^3^, ^5^, ^7^ Further research is urgently needed to harness the immune response and develop effective vaccines against SARS‐CoV‐2.

Here, we show a possible association between HLA‐A*02:01 and an increased risk for COVID‐19. We first focused on the global distribution of HLA gene polymorphism and examined the association of the most frequent HLA alleles in countries with prevalence and mortality of COVID‐19. Then, we employed computational in silico binding prediction of HLA class I alleles for SARS‐CoV‐2 peptides. HLA‐A*02:01 is predicted to have a relatively lower capacity to present SARS‐CoV‐2 antigens compared with other frequent HLA class I molecules (HLA‐A*11:01 or HLA‐A*24:02), suggesting that individuals with HLA‐A*11:01 or HLA‐A*24:02 genotypes may generate efficiently T‐cell‐mediated antiviral responses to SARS‐CoV‐2 compared with HLA‐A*02:01. The differences in HLA genotypes may potentially alter the course of the disease and its transmission. Our study suggests that identifying the HLA genotypes associated with the severity of COVID‐19 or susceptibility to SARS‐CoV‐2 may support future vaccination strategies to genotypically at‐risk populations.

## 2 MATERIALS AND METHODS

### 2.1 HLA allele frequency analysis in 19 countries

Nineteen countries with high or low mortality for COVID‐19 as of April 24, 2020 were selected. Frequencies of HLA class I alleles (HLA‐A, ‐B, and ‐C) and HLA class II alleles (HLA‐DPB1, ‐DQB1, and ‐DRB1) in different 19 countries were searched by a publicly available database, Allele Frequency Net Database (http://www.allelefrequencies.net), using an HLA searching option (HLA classical allele freq search). The most frequent HLA genotypes were selected for each country (Tables 1 and 2). HLA frequency data from a race, which consists of the majority of the population in each country, were used for the analysis. HLA frequency data from minorities of the population were excluded from the analysis. There was not enough data of HLA frequency for Canada, New Zealand, and the Russian Federation; thus these three countries were searched, but not included in the analysis.

**Table 1:** Common HLA alleles in 19 countries, confirmed cases and deaths caused by COVID‐19 as of April 24, 2020

Country	Australia	Belgium	Brazil	China	France	Germany	India	Iran	Israel	Italy	Japan	Mexico	Singapore	South Korea	Spain	Thailand	Turkey	England	U.S.A.
HLA‐A*	02:01	02:01	02:01	11:01	02:01	02:01	24:02	24:02	02:01	02:01	24:02	02:01	11:01	24:02	02:01	11:01	02:01	02:01	02:01
HLA‐B*	08:01	08:01	08:01	13:02	08:01	07:02	40:06	35:01	35:01	51:01	52:01	15:01	40:01	15:01	18:01	46:01	N/A	07:02	07:02
HLA‐C*	07:01	N/A	07:01	01:02	07:01	07:01	06:02	04:01	N/A	07:01	01:02	07:02	07:02	01:02	04:01	07:01	07:01	07:01	07:01
HLA‐DPB1*	N/A	N/A	N/A	05:01	04:01	04:01	04:01	N/A	N/A	04:01	05:01	04:01	05:01	05:01	04:01	05:01	N/A	04:01	04:01
HLA‐DQB1*	N/A	04:01	03:01	03:01	03:01	03:01	02:01	03:01	03:01	03:01	06:01	03:02	06:01	03:01	03:01	05:02	03:01	02:01	03:01
HLA‐DRB1*	N/A	03:01	07:01	07:01	04:01	15:01	07:01	15:01	11:04	07:01	09:01	08:02	04:06	09:01	07:01	15:02	11:01	15:01	15:01
Confirmed cases	276	3768	220	60	1848	1835	17	1084	1807	7874	97	83	1988	211	4596	41	1280	2099	2576
Deaths	3.2	571	14.0	3.3	337	65.0	0.5	68.3	23.4	1059	2.5	7.6	2.1	4.7	478	0.7	31.3	285	131

*Note*: Nineteen countries with high or low mortality for COVID‐19 were selected. Frequencies of three HLA class I alleles (A, B, and C) and three HLA class II alleles (DPB1, DQB1, and DRB1) in different 19 countries were searched by a publicly available database (Allele Frequency Net Database, http://www.allelefrequencies.net). The most frequent HLA alleles are selected for each country. HLA frequency data from a race, which consists of the majority of each country, were used for analysis. For all countries, the HLA frequency data of minorities were excluded from the analysis. There was not enough data for Canada and New Zealand. Total confirmed cases per million population and total deaths per million population for COVID‐19 are indicated. Data on COVID‐19 cases and death per country were obtained from the World Health Organization (WHO) coronavirus disease (COVID‐2019) situation reports (https://www.who.int/emergencies/diseases/novel-coronavirus-2019/situation-reports) on April 24, 2020. Data of the total population of countries were obtained from the WHO website (https://www.who.int/countries/en/).

Abbreviations: Abbreviations: HLA, human leukocyte antigen; N/A, not applicable.

**Table 2:** Common HLA alleles in 19 countries, confirmed cases and deaths caused by COVID‐19 as of August 15, 2020

Country	Australia	Belgium	Brazil	China	France	Germany	India	Iran	Israel	Italy	Japan	Mexico	Singapore	South Korea	Spain	Thailand	Turkey	England	U.S.A.
HLA‐A*	02:01	02:01	02:01	11:01	02:01	02:01	24:02	24:02	02:01	02:01	24:02	02:01	11:01	24:02	02:01	11:01	02:01	02:01	02:01
HLA‐B*	08:01	08:01	08:01	13:02	08:01	07:02	40:06	35:01	35:01	51:01	52:01	15:01	40:01	15:01	18:01	46:01	N/A	07:02	07:02
HLA‐C*	07:01	N/A	07:01	01:02	07:01	07:01	06:02	04:01	N/A	07:01	01:02	07:02	07:02	01:02	04:01	07:01	07:01	07:01	07:01
HLA‐DPB1*	N/A	N/A	N/A	05:01	04:01	04:01	04:01	N/A	N/A	04:01	05:01	04:01	05:01	05:01	04:01	05:01	N/A	04:01	04:01
HLA‐DQB1*	N/A	04:01	03:01	03:01	03:01	03:01	02:01	03:01	03:01	03:01	06:01	03:02	06:01	03:01	03:01	05:02	03:01	02:01	03:01
HLA‐DRB1*	N/A	03:01	07:01	07:01	04:01	15:01	07:01	15:01	11:04	07:01	09:01	08:02	04:06	09:01	07:01	15:02	11:01	15:01	15:01
Confirmed cases	943	6781	15,530	64	3073	2720	1908	4220	10,801	10,479	419	3965	9886	296	73,965	48	3105	4809	16,150
Deaths	16	874	508	3.3	468	113	37	240	78	1460	8.5	434	4.8	6.0	617	0.8	75	629	515

*Note*: Nineteen countries with high or low mortality for COVID‐19 were selected. Frequencies of three HLA class I alleles (A, B, and C) and three HLA class II alleles (DPB1, DQB1, and DRB1) in different 19 countries were searched by a publicly available database (Allele Frequency Net Database, http://www.allelefrequencies.net). The most frequent HLA alleles are selected for each country. HLA frequency data from a race, which consists of the majority of each country, were used for analysis. For all countries, HLA frequency data of minorities were excluded from the analysis. There was not enough data for Canada and New Zealand. Total confirmed cases per million population and total deaths per million population for COVID‐19 are indicated. Data on COVID‐19 cases and death per country were obtained from the World Health Organization (WHO) coronavirus disease (COVID‐2019) situation reports (https://www.who.int/emergencies/diseases/novel-coronavirus-2019/situation-reports) on August 15, 2020. Data on the total population of countries were obtained from the WHO website (https://www.who.int/countries/en/).

Abbreviations: HLA, human leukocyte antigen; N/A, not applicable.

### 2.2 COVID‐19 cases and deaths per country

Data on COVID‐19 cases and death per country were obtained from the World Health Organization (WHO) coronavirus disease (COVID‐2019) situation reports (https://www.who.int/emergencies/diseases/novel-corona-virus-2019/situation-reports) on April 24, 2020 and on August 15, 2020. Data on the total population of countries were obtained from the WHO website (https://www.who.int/countries/en/). Total confirmed cases per million population and total deaths per million population for COVID‐19 were calculated for each country (Tables 1 and 2).

### 2.3 SARS‐CoV‐2 CD8^+^ T‐cell epitope prediction

We employed computational in silico binding prediction of the HLA class I alleles for SARS‐CoV‐2 peptides. We aimed to calculate the total number of SARS‐CoV‐2 peptides, which HLA class I alleles are predicted to bind. To calculate the number of bound peptides by each allele we used the HLA‐peptide binding prediction algorithms, the Immune Epitope Database and Analysis Resource (IEDB; T‐cell epitope prediction tool, Prediction Method Version 2.23, http://tools.iedb.org/main/tcell/). Epitope prediction was carried out using the reference SARS‐CoV‐2 isolate, Wuhan‐Hu‐1. Protein sequences from the entire SARS‐CoV‐2 proteome were obtained from the SARS‐CoV‐2 reference sequence (GenBank: MN908947.3).^10^ The HLA class I molecules typically bind peptides 8–11 amino acids.^6^ Thus, HLA binding affinity of all possible 8‐mer to 11‐mer across the entire SARS‐CoV‐2 proteome was assessed. The SARS‐CoV‐2 protein sequences were run against HLA alleles using the NetMHCpan EL 4.0 algorithm available at the IEDB (http://tools.iedb.org/mhci/) and a size range of 8–11‐mers.^11^, ^12^ Eight to eleven contiguous amino acids sliding one amino acid residue were taken through the full sequence of the entire SARS‐CoV‐2 proteome. Top 0.5%, 0.5% < percentile rank ≤ 1%, and 1% < percentile rank ≤ 2% epitopes ranked based on prediction score (high to low predicted binding affinity to HLA‐A*02:01, HLA‐A*11:01, or HLA‐A*24:02), were selected for each HLA class I allele analyzed^12^ and the total numbers of predicted 8–11‐mer SARS‐CoV‐2 T‐cell epitopes per HLA class I genotype were counted.

### 2.4 Statistical analysis

The differences of deaths per 10^6^ population and confirmed cases per 10^6^ population for COVID‐19 between countries in which HLA‐A*02:01 is the most frequent in the population (HLA‐A*02:01 group) and countries in which other HLA genotypes (HLA‐A*11:01 and HLA‐A*24:02) are the most frequent in the population (non‐HLA‐A*02:01 group) were evaluated by permuted Brunner–Munzel test.^13^ Spearman's correlation coefficients and simple linear regression model were employed to examine the relationship between deaths per 10^6^ population and confirmed cases per 10^6^ population for COVID‐19 according to HLA genotypes. Moreover, the difference of deaths per 10^6^ population between HLA‐A*02:01 and non‐HLA‐A*02:01 groups was employed by analysis of covariance (ANCOVA) based on confirmed cases per 10^6^ population for COVID‐19. Analyses were performed using R version 3.5.2 (The R Foundation for Statistical Computing) with the level of statistical significance set at *p* < .05.

## 3 RESULTS

### 3.1 HLA genotypes may impact on the differences in morbidity and mortality of COVID‐19 across countries

We hypothesized that the most common HLA genotypes in the population might affect the differences in morbidity and mortality of COVID‐19 among countries. We first focused on the global distribution of HLA gene polymorphism. To evaluate our hypothesis, we examined the association of the most frequent HLA alleles in countries with prevalence and mortality of COVID‐19 by using publicly available databases. The HLA frequency data from 19 countries with high or low mortality for COVID‐19 were analyzed. We searched the frequencies of HLA class I alleles (HLA‐A, ‐B, and ‐C) and HLA class II alleles (HLA‐DPB1, ‐DQB1, and ‐DRB1) in 19 countries by a publicly available database (Allele Frequency Net Database, http://www.allelefrequencies.net). Then, the most common HLA alleles were selected for each country (Tables 1 and 2).

Among the frequent HLA genotypes, we found that the most frequent HLA alleles, HLA‐A*02:01, ‐C*07:01, ‐DPB1*04:01, and ‐DQB1*03:01, are shared by more than half of the countries. Thus, we focused on these HLA genotypes and divided the 19 countries into two groups: countries in which these HLA genotypes are the most frequent in the population versus countries in which other HLA genotypes are the most frequent in the population. Then, we examined the association of the most frequent HLA genotypes with the total confirmed cases per million population and total deaths per million population for COVID‐19 (Tables 1 and 2).

**Figure 1:**
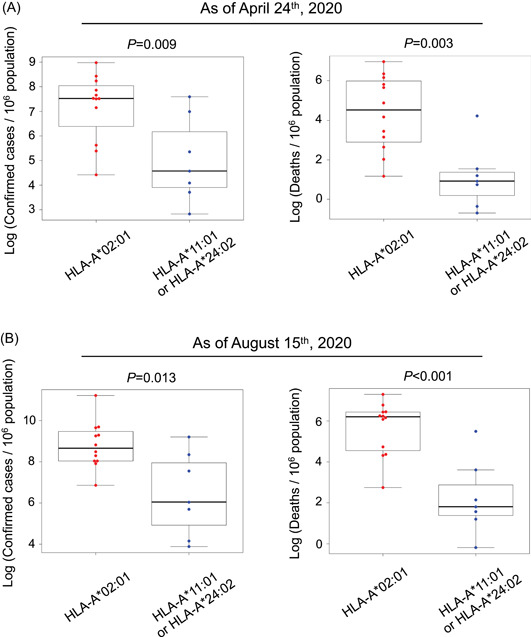
Association of human leukocyte antigen (HLA) class I gene polymorphisms with prevalence and mortality of COVID‐19. (A) Differences in total confirmed cases and deaths caused by COVID‐19 between countries in which HLA‐A*02:01 is the most frequent in the population (HLA‐A*02:01 group) and countries in which other HLA genotypes (HLA‐A*11:01 and HLA‐A*24:02) are the most frequent in the population (non‐HLA‐A*02:01 group) as of April 24, 2020. (B) Differences in total confirmed cases and deaths caused by COVID‐19 between HLA‐A*02:01 and non‐HLA‐A*02:01 groups as of August 15, 2020. Permuted Brunner–Munzel test was performed

Although HLA‐C, ‐DPB1, or ‐DQB1 alleles did not show significant differences between countries in total confirmed cases and deaths caused by COVID‐19, HLA‐A allele showed noticeable results (Figure 1). As of April 24, 2020, the countries in which HLA‐A*02:01 is the most frequent in the population (HLA‐A*02:01 group) had total confirmed cases with median 1842 cases per million population. In contrast, the countries in which other HLA genotypes, HLA‐A*24:02 or HLA‐A*11:01, are the most frequent in the population (non‐HLA‐A*02:01 group) had lower total confirmed cases with median 97 cases per million population. This difference between HLA‐A*02:01 and non‐HLA‐A*02:01 groups was significant (*p* = .01; permuted Brunner–Munzel test; Figure 1A, left panel). Similarly, HLA‐A*02:01 is significantly correlated with an increase of total deaths per million population caused by COVID‐19 (HLA‐A*02:01 vs. HLA‐A*24:02 or HLA‐A*11:01; median, 98 vs. 2.5; *p* = .003; Figure 1A, right panel). We also analyzed the updated data of morbidity and mortality of COVID‐19 as of August 15, 2020. We confirmed that HLA‐A*02:01 is significantly associated with an increase of total COVID‐19 confirmed cases per million population (HLA‐A*02:01 vs. HLA‐A*24:02 or HLA‐A*11:01; median, 5795 vs. 419; *p* = .013; Figure 1B, left panel) and total deaths per million population caused by COVID‐19 (HLA‐A*02:01 vs. HLA‐A*24:02 or HLA‐A*11:01; median, 488 vs. 6.1; *p* < .001; Figure 1B, right panel).

**Figure 2:**
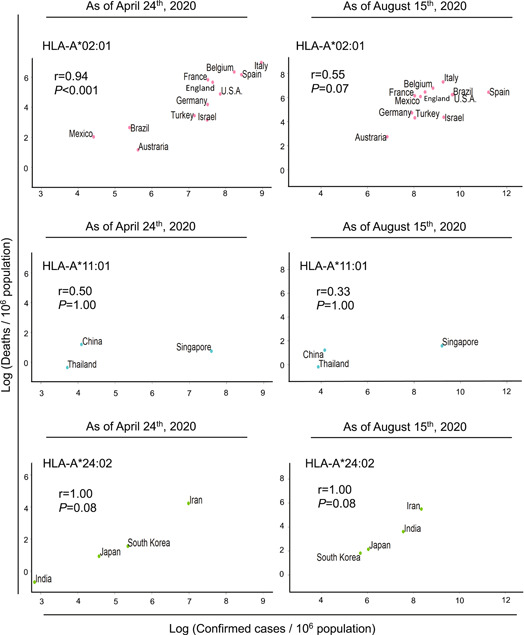
A relationship between deaths per 10^6^ population, confirmed COVID‐19 cases per 10^6^ population, and the most common HLA‐A genotypes in each country. Upper panels indicate that the relationship between deaths confirmed, COVID‐19 cases, and countries in which HLA‐A*02:01 is the most frequent in the population as of April 24 (left panel) and August 15, 2020 (right panel). Pink dots indicate the distribution of Australia, Belgium, Brazil, France, Germany, Mexico, Spain, Turkey, England, U.S.A., Israel, and Italy. Middle panels indicate that the relationship between deaths, confirmed COVID‐19 cases, and countries in which HLA‐A*11:01 is the most frequent in the population. Blue dots indicate the distribution of China, Singapore, and Thailand. Lower panels indicate that the relationship between deaths, confirmed COVID‐19 cases, and countries in which HLA‐A*24:02 is the most frequent in the population. Green dots indicate the distribution of India, Iran, Japan, and South Korea. Spearman's correlation coefficients and the *p* value were denoted as “r” and “*P*,” respectively. COVID‐19, coronavirus disease 2019; HLA, human leukocyte antigen

To assess the association between deaths and confirmed cases for COVID‐19, according to HLA genotypes, we used Spearman's correlation analysis. In HLA‐A*02:01 group, deaths for COVID‐19 showed a trend toward increase according to the rise in confirmed cases as of  April 24 and August 15, 2020 (Figure 2). The association between death per 10^6^ population, confirmed cases per 10^6^ population, and HLA class I genotypes (HLA‐A*02:01, HLA‐A*11:01, and HLA‐A*24:02) are shown in Figure 2. The results of the univariate regression analysis also showed similar results (Table S1).

To exclude the possibility that the number of deaths caused by COVID‐19 was simply increased according to an increase of confirmed cases of COVID‐19 regardless of HLA genotypes, we next performed ANCOVA and adjusted the number of confirmed cases of COVID‐19 between HLA‐A*02:01 and non‐HLA‐A*02:01 groups. Accordingly, the confirmed cases of COVID‐19 as of August 15, 2020 was associated with an increase of deaths in HLA‐A*02:01 (*p* = .005; 95% confidence interval [CI]: 0.25–1.01; Table 3). Moreover, our analysis showed an increase of deaths caused by COVID‐19 in HLA‐A*02:01 group compared with non‐HLA‐A*02:01 group (*p* = .020; 95% CI: 0.46–3.37; Table 3). These results suggest a possible association between HLA‐A*02:01 and an increased risk for COVID‐19. Data as of April 24, 2020 are shown in Table S2. Geographical frequent HLA allele distribution for HLA‐A*02:01, HLA‐A*24:02, and HLA‐A*11:01 among the 19 countries is shown in Supplementary Figure 1.

**Table 3:** Results of ANCOVA as of August 15, 2020

Endogenous variable	Exogenous variable	Estimate	*SE*	*p* value	95% CI
Log (deaths)	Intercept	−1.80	1.33	.19	−4.41, 0.81
	Log (confirmed cases)	0.63	1.94	.005	0.25, 1.01
	non‐HLA‐A*02:01 (Ref.)				
	HLA‐A*02:01	1.91	0.74	.020	0.46, 3.37

*Note*: Total confirmed cases per million population (confirmed cases) and total deaths per million population (deaths) for COVID‐19 are calculated.

Abbreviations: ANCOVA, analysis of covariance; CI, confidence interval; HLA, human leukocyte antigen; Ref., reference.

### 3.2 HLA‐A*02:01 may have a lower capacity to present SARS‐CoV‐2 antigens compared with HLA‐A*11:01 and HLA‐A*24:02

**Figure 3:**
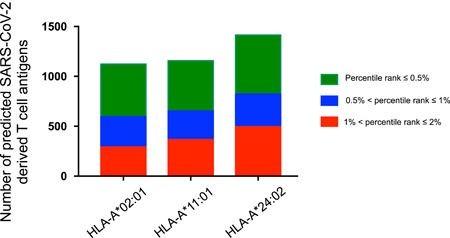
Number of predicted SARS‐CoV‐2‐derived T‐cell antigens. A comparison of the numbers of predicted SARS‐CoV‐2‐derived T‐cell antigens between HLA‐A*02:01, HLA‐A*11:01, or HLA‐A*24:02 is shown. Epitope prediction was carried out using the reference SARS‐CoV‐2 isolate, Wuhan‐Hu‐1. Protein sequences from the entire SARS‐CoV‐2 proteome were obtained from the SARS‐CoV‐2 reference sequence (GenBank: MN908947.3). HLA binding affinity of all possible 8–11‐mer across the entire SARS‐CoV‐2 proteome was assessed. The SARS‐CoV‐2 protein sequences were run against HLA alleles using the NetMHCpan EL 4.0 algorithm available at the IEDB (http://tools.iedb.org/mhci/) and a size range of 8–11‐mers. Top 0.5% (red), 0.5% < percentile rank ≤ 1% (blue), and 1% < percentile rank ≤ 2% (green panel) epitopes ranked based on prediction score (high to low predicted binding affinity to HLA‐A*02:01, HLA‐A*11:01, or HLA‐A*24:02) were selected and the total number of predicted 8–11‐mer SARS‐CoV‐2 T‐cell epitopes per HLA class I genotype were counted. HLA, human leukocyte antigen; IEDB, Immune Epitope Database and Analysis Resource; SARS‐CoV‐2, severe acute respiratory syndrome coronavirus 2

Next, we hypothesized that these HLA class I genotypes (HLA‐A*02:01, HLA‐A*11:01, and HLA‐A*24:02) may differentially generate T‐cell‐mediated antiviral response and could potentially alter the course of the disease and its transmission. To explore the potential for an HLA allele to produce an antiviral response, we assessed the HLA binding affinity of possible 8–11‐mer from the entire SARS‐CoV‐2 proteome (MN908947.3). We performed an in silico analysis of HLA class I binding predictions using the Net MHC pan 4.0 EL algorithm available at the IEDB.^11^ For each HLA class I allele analyzed, we selected the top 0.5%, 1%, and 2% scoring peptides in the SARS‐CoV‐2 sequence, as ranked based on prediction.^11^, ^12^ The total numbers of possible SARS‐CoV‐2 antigens, which have high to low binding affinity to HLA‐A*02:01, HLA‐A*11:01, or HLA‐A*24:02, were counted. Interestingly, both HLA‐A*11:01 and HLA‐A*24:02 had a relatively larger capacity to present SARS‐CoV‐2 antigens compared with HLA‐A*02:01 (Figure 3). Although HLA‐A*11:01 had a relatively larger capacity to present SARS‐CoV‐2 antigens compared with HLA‐A*02:01 only in peptides predicted high binding affinity, HLA‐A*24:02 was predicted to have the largest capacity to present SARS‐CoV‐2‐derived antigens, which have high to low binding affinity (Figures 3 and S2). These results suggest that individuals with HLA‐A*11:01 and HLA‐A*24:02 genotypes may generate efficiently T‐cell‐mediated antiviral responses to SARS‐CoV‐2 compared with HLA‐A*02:01, and could potentially alter the course of the disease and its transmission.

## 4 DISCUSSION

We propose a hypothesis that HLA genotypes may impact the differences in morbidity and mortality of COVID‐19 across countries based on our preliminary data from in silico analyses, which indicated a possible association between HLA‐A*02:01 and an increased risk for COVID‐19 due to lower capacity to present SARS‐CoV‐2 antigens.

HLA class I is a critical component of the viral antigen presentation pathway and confer differential viral susceptibility and severity of the disease.^14^, ^15^ A virus infects cells directly through the interactions between virus particles and their receptors at the cell surface. After a virus infects a cell, the virus uses the protein synthesis machinery of the host cell to synthesize its own proteins.^14^, ^15^ Some of the synthesized viral proteins are degraded into 8–11‐mer peptide fragments, which bind to HLA class I molecules if they have sufficient binding affinity. Then, the HLA class I peptide complex is presented on the cell surface of an infected cell. Viral specific CD8^+^ T cells can recognize the HLA class I peptide complex and induce apoptosis of the infected cell. Therefore, CD8^+^ T cells control viral infection through its direct cytotoxic activity and pro‐inflammatory cytokine productions. Recent studies have shown that SARS‐CoV‐2‐specific CD8^+^ T cells can be detected in peripheral blood mononuclear cells of COVID‐19 patients,^16^, ^17^, ^18^, ^19^ suggesting that some SARS‐CoV‐2‐derived peptides can be naturally processed in human cells, presented on HLA class I molecules and induce SARS‐CoV‐2‐specific cytotoxic CD8^+^ T cells in vivo. Accumulating evidence highlights the importance of investigating associations between HLA variation and the development and/or severity of COVID‐19.^7^, ^8^, ^9^

In a recent study of in silico binding affinity predictions across 145 different HLA‐A, ‐B, and ‐C alleles, Nguyen et al.^7^ have identified that HLA‐B*46:01 as the HLA allele with the fewest predicted binding peptides for SARS‐CoV‐2. To assess the potential for cross‐protective immunity conferred by prior exposure to common human coronaviruses, the study focused on 48 highly conserved amino acid sequence spans among all known human coronaviruses. For these potentially cross‐protective peptides, binding affinity predictions across 145 different HLA‐class I alleles were performed. Then, HLA‐A*02:02, HLA‐B*15:03, and HLA‐C*12:03 alleles were found as the top presenters of conserved peptides. Among the three HLA‐class I alleles, HLA‐B*15:03 has been shown to have the greatest capacity to present highly conserved SARS‐CoV‐2 peptides that are shared among common human coronaviruses.^7^

The severe COVID‐19 genome‐wide association study (GWAS) group have conducted a genome‐wide association study involving 835 patients with COVID‐19 and 1255 control participants from Italy and 775 patients with COVID‐19 and 950 control participants from Spain.^5^ In this study, extensive analysis of the classical HLA loci showed no significant allele associations with either COVID‐19 or disease severity in Spain and Italy. In addition, this study reported that analyses of heterozygote and divergent allele advantage or the predicted number of HLA‐bound SARS‐CoV‐2 peptides did not show significant associations with COVID‐19.^5^ Wang et al. have studied HLA allele frequencies in Chinese Han individuals with COVID‐19. An association between HLA allele distribution in COVID‐19 patients and healthy individuals was assessed.^9^ In this study, 82 patients with COVID‐19 were genotyped for HLA‐A, ‐B, ‐C, ‐DRB1, ‐DRB3/4/5, ‐DQA1, ‐DQB1, ‐DPA1, and ‐DPB1 loci using next‐generation sequencing. This study reported that frequencies of the HLA‐C*07:29 and HLA‐B*15:27 alleles were higher in COVID‐19 patients than those in the control population. Two different studies from Wang et al.^9^ and the severe COVID‐19 GWAS group were conducted in different countries, which might have led to different results.^5^

In our study, we focused on the global distribution of HLA gene polymorphism. We hypothesized that the most common HLA genotypes in the population might affect the differences in morbidity and mortality of SARS‐CoV‐2 among countries. Therefore, we first searched the frequencies of HLA class I and HLA class II alleles in different 19 countries and selected the most common HLA alleles for each country. Among the frequent HLA genotypes, we found that HLA‐A*02:01, ‐C*07:01, ‐DPB1*04:01, and ‐DQB1*03:01 are shared by more than half of the countries. Thus, we focused on these HLA alleles and divided the 19 countries into two groups and then compared morbidity/mortality of SARS‐CoV‐2 between these two groups. Finally, we found a possible association between HLA‐A*02:01 and an increased risk for COVID‐19. Our analyses showed different results from recent studies.^5^, ^7^, ^9^ We took different approaches from previous studies to assess the association between HLA genotypes and COVID‐19, which might be one of the reasons why different results were obtained in the current study. To resolve these discrepancies, further studies at a global level are urgently required.

More than 15,000 different classical HLA class I and II alleles have been identified.^6^ The diversity of HLA molecules and the complex HLA haplotypes at the population level may buffer against the lack of presentation from a single poorly presenting allele and can mount an immune attack against COVID‐19. In the current study, we did not assess the impact of frequent HLA haplotypes on the prevalence and mortality of COVID‐19, which needs to be assessed in future studies.

This study has limitations in view of the retrospective nature. The results of the current study may be affected by unknown confounding factors. There are many factors that could potentially impact on morbidity and mortality for COVID‐19: geographical, social, and biological factors such as temperature, humidity, life expectancy, age, comorbidity, average income, social norms, social distancing measures, face mask wearing rate, BCG vaccination policy, genetic factors other than HLA, and the time since the onset of COVID‐19 epidemic.^2^, ^3^, ^5^, ^7^, ^9^, ^20^ These all factors may serve as a potentially confounding factor. These elements might affect the observed relationships between COVID‐19 prevalence/mortality and common HLA genotypes by affecting both the susceptibility to SARS‐CoV‐2 and population characteristics in an indirect manner. However, we did not address these possible confounding factors. We explored only a limited set of data from selected countries. In addition, the results shown in the current study entirely originated from in silico analyses. In vitro analysis of SARS‐CoV‐2‐specific T‐cell induction and cytotoxic efficiency of the induced CD8^+^ T cells need to be investigated by using SARS‐CoV‐2 protein and peripheral blood mononuclear cells from healthy donors with HLA‐A*02:01, HLA‐A*11:01, or HLA‐A*24:02. Genotypic heterogeneity or in vivo evolution of SARS‐CoV‐2 could modify the repertoire of viral epitopes presented by HLA,^7^, ^21^ which we did not assess in the current study. Hoffmann et al.^22^ have shown that SARS‐CoV‐2 uses the SARS‐CoV receptor angiotensin‐converting enzyme 2 (ACE2) for entry and the serine protease transmembrane serine protease 2 (TMPRSS2) for S protein priming, indicating SARS‐CoV‐2 infection depends on the host cell factors ACE2 and TMPRSS2. These studies suggest that host genetic variation could modulate the host‐pathogen interface in an HLA‐independent manner. However, we also did not assess these possibilities in this study. Therefore, our findings should be interpreted with caution and our hypothesis needs to be further investigated. Although we are unable to obtain HLA genotype information for patients with COVID‐19 at this time, our hypothesis proposed here could be clinically validated by integrating HLA typing into prospective COVID‐19 clinical studies. Prospective studies are urgently needed at a global level.

Harnessing the HLA function clinically is a challenging mission.^4^, ^6^, ^7^, ^8^ The progress in characterizing HLA diversity and HLA associations with human disease improved our understanding of HLA function for clinical benefit.^6^ It is considered that the capacity to stratify patients based on their HLA genotypes and to administer antigen‐specific, patient‐tailored disease prevention, presents an especially attractive target with the current aim of achieving precision or personalized medicine.^6^, ^7^ Identifying the HLA genotype associated with the severity of COVID‐19 or susceptibility to SARS‐CoV‐2 may support future vaccination strategies to genotypically at‐risk populations.^6^, ^7^ Individuals with high‐risk HLA types could be prioritized for vaccines against SARS‐CoV‐2, which may lead to effectively reduce the COVID‐19 morbidity and mortality.^7^, ^10^ Our study may provide new insights into the COVID‐19 pandemic and improve our understanding of host immune response against SARS‐CoV‐2.

## CONFLICT OF INTERESTS

The authors declare that there are no conflict of interests.

## ETHICS STATEMENT

The present study was approved by the Kumamoto University Institutional Review Board (IRB number, 2020; approval date, April 17, 2020). This study was prepared in accordance with the Helsinki Declaration.

## AUTHOR CONTRIBUTIONS

*Conception and design*: Yusuke Tomita and Tokunori Ikeda. *Acquisition of data*: Yusuke Tomita, Tokunori Ikeda, and Ryo Sato. *Analysis and interpretation of data*: Yusuke Tomita, Tokunori Ikeda, and Ryo Sato. *Study supervision*: Yusuke Tomita, Tokunori Ikeda, and Takuro Sakagami. *Writing, review, and/or revision of the manuscript*: All authors.

## Supporting information

Supporting information.

Supporting information.

Supporting information.

Supporting information.

Supporting information.

## Data Availability

The data that support the findings of this study are available from the corresponding author upon reasonable request.
