# Supplementary material for: Association between HLA gene polymorphisms and mortality of COVID‐19: An in silico analysis
Source: Immun Inflamm Dis. 2020 Oct 13;8(4):684–94. doi: 10.1002/iid3.358 (PMC7654404; doi:10.1002/iid3.358)
Supplement: Supplementary file 1 — Supporting information. [file IID3-8-684-s001.pdf]

Supplementary Figure 1

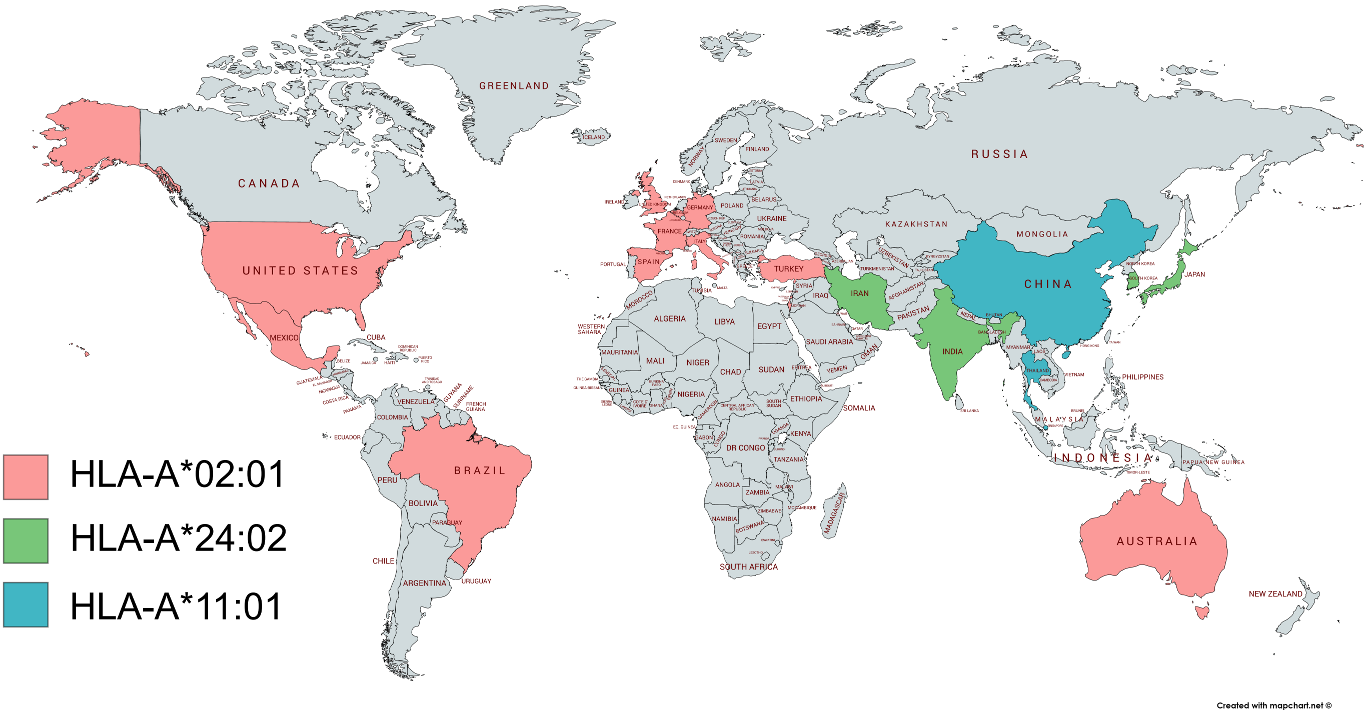

Supplementary Figure Legend

Supplementary Figure S1. Global HLA allele distribution for HLA-A*02:01, -A*11:01 and -A*24:02. World map depicting distribution of HLA-A*02:01, -A*11:01, and -A*24:02 alleles was created with mapchart.net.
