# Supplementary material for: Association between HLA gene polymorphisms and mortality of COVID‐19: An in silico analysis
Source: Immun Inflamm Dis. 2020 Oct 13;8(4):684–94. doi: 10.1002/iid3.358 (PMC7654404; doi:10.1002/iid3.358)
Supplement: Supplementary file 2 — Supporting information. [file IID3-8-684-s002.pdf]

Supplementary Figure S2

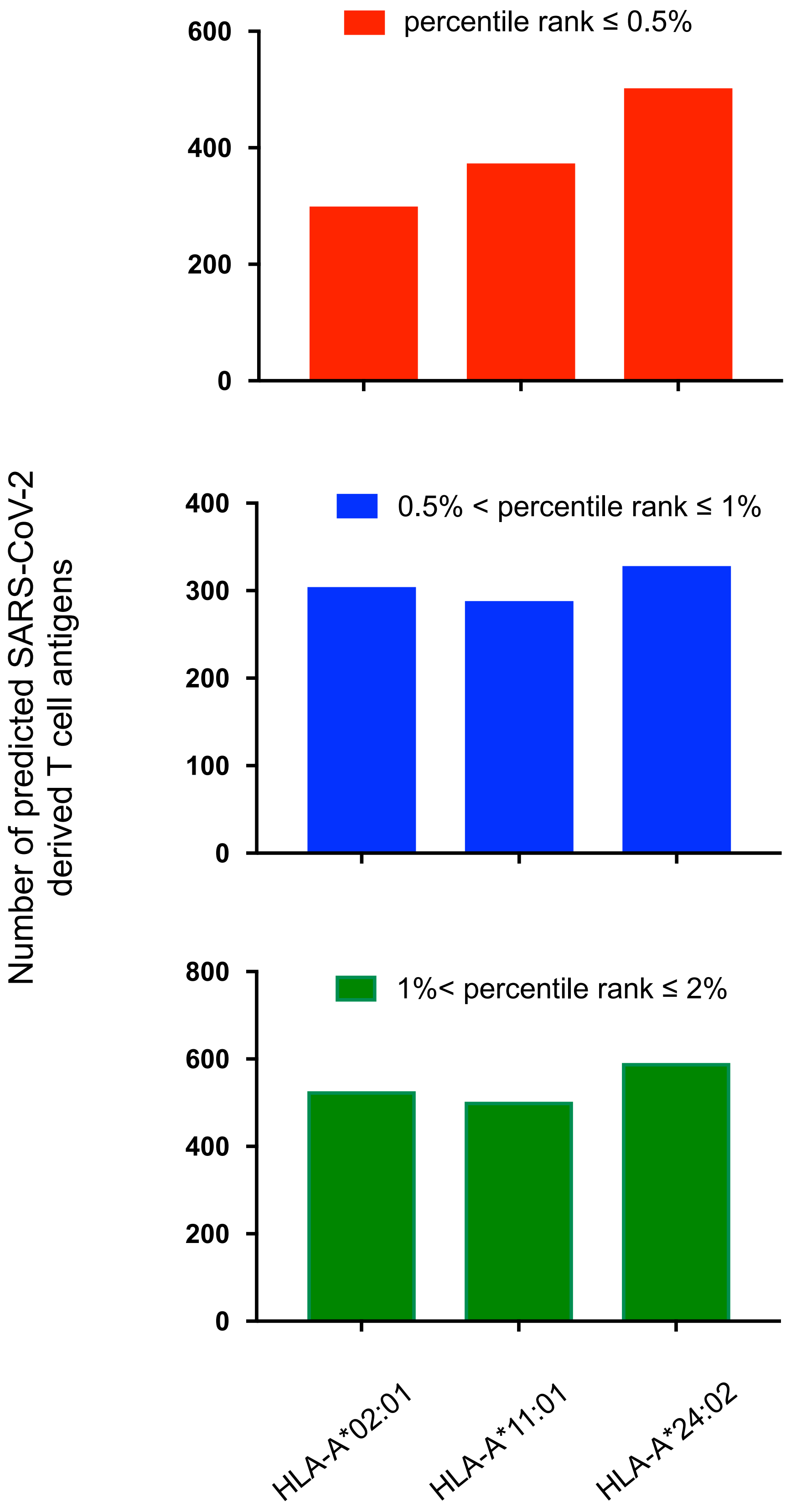

Supplementary Figure S2 Legend

Supplementary Figure S2. Number of predicted SARS-CoV2-derived T cell antigens. Comparisons of the numbers of predicted SARS-CoV2-derived T cell antigens between HLA-A*02:01, HLA-A*11:01 or HLA-A*24:02 are shown. For each HLA class I allele analyzed, we selected the top 0.5% (upper panel), 0.5% < percentile rank ≤ 1% (middle panel) and 1%< percentile rank ≤ 2% (lower panel) scoring peptides in the SARS-CoV-2 sequence, as ranked based on prediction. Top 0.5%, 1% and 2% epitopes ranked based on prediction score (high to low predicted binding affinity to HLA-A*02:01, HLA-A*11:01 or HLA-A*24:02), were selected for each HLA class I allele analyzed. The SARS-CoV-2 protein sequences were run against HLA alleles using the NetMHCpan EL 4.0 algorithm available at the IEDB (http://tools.iedb.org/mhci/) and a size range of 8-11mers.
