# Supplementary material for: Association between HLA gene polymorphisms and mortality of COVID‐19: An in silico analysis
Source: Immun Inflamm Dis. 2020 Oct 13;8(4):684–94. doi: 10.1002/iid3.358 (PMC7654404; doi:10.1002/iid3.358)
Supplement: Supplementary file 3 — Supporting information. [file IID3-8-684-s003.docx]

**Supplementary Table S1. Results of univariate regression analysis as of April 24^th^, 2020.**

| **Endogenous variable** | **Exogenous variable** | **Estimate** | **SE** | ***p*-Value** | **95% CI** |
| --- | --- | --- | --- | --- | --- |
| **HLA-A*02:01** |  |  |  |  |  |
| Log (Deaths) | Intercept | -4.39 | 1.55 | 0.018 | (−7.85, -0.94) |
|  | Log (Confirmed cases) | 1.22 | 0.21 | <0.001 | (0.75, 1.69) |
| **HLA-A*11:01** |  |  |  |  |  |
| Log (Deaths) | Intercept | -0.09 | 1.92 | 0.97 | (−24.42, 24.25) |
|  | Log (Confirmed cases) | 0.12 | 0.35 | 0.79 | (-4.37, 4.61) |
| **HLA-A*24:02** |  |  |  |  |  |
| Log (Deaths) | Intercept | -4.26 | 0.77 | 0.031 | (−7.56, -0.97) |
|  | Log (Confirmed cases) | 1.17 | 0.15 | 0.016 | (0.53, 1.81) |

**Results of univariate regression analysis (as of August 15^th^, 2020).**

| **Endogenous variable** | **Exogenous variable** | **Estimate** | **SE** | ***P*-Value** | **95% CI** |
| --- | --- | --- | --- | --- | --- |
| **HLA-A*02:01** |  |  |  |  |  |
| Log (Deaths) | Intercept | -0.48 | 2.63 | 0.86 | (−6.34, 5.39) |
|  | Log (Confirmed cases) | 0.70 | 0.30 | 0.041 | (0.03, 1.36) |
| **HLA-A*11:01** |  |  |  |  |  |
| Log (Deaths) | Intercept | -0.37 | 1.39 | 0.84 | (−18.01, 17.27) |
|  | Log (Confirmed cases) | 0.21 | 0.22 | 0.51 | (-2.61, 3.04) |
| **HLA-A*24:02** |  |  |  |  |  |
| Log (Deaths) | Intercept | -5.76 | 1.40 | 0.05 | (−11.78, 0.26) |
|  | Log (Confirmed cases) | 1.31 | 0.20 | 0.023 | (0.45, 2.17) |

Abbreviations: SE, Standard error; CI, Confidence interval. Total confirmed cases per million population (Confirmed cases) and total deaths per million population (Deaths) for COVID-19 are calculated.
