# Supplementary material for: Association between HLA gene polymorphisms and mortality of COVID‐19: An in silico analysis
Source: Immun Inflamm Dis. 2020 Oct 13;8(4):684–94. doi: 10.1002/iid3.358 (PMC7654404; doi:10.1002/iid3.358)
Supplement: Supplementary file 4 — Supporting information. [file IID3-8-684-s004.docx]

**Supplementary Table S2.** Results of analysis of covariance (ANCOVA) as of April 24^th^, 2020.

| **Endogenous variable** | **Exogenous variable** | **Estimate** | **SE** | ***p*-Value** | **95% CI** |
| --- | --- | --- | --- | --- | --- |
| Log (Deaths) | Intercept | -3.61 | 1.03 | 0.003 | (−5.62, -1.59) |
|  | Log (Confirmed cases) | 0.93 | 0.19 | <0.001 | (0.57, 1.30) |
|  | non-HLA-A*02:01 (Ref.) |  |  |  |  |
|  | HLA-A*02:01 | 1.26 | 0.68 | 0.08 | (-0.07, 2.59) |

Abbreviations: SE, Standard error; CI, Confidence interval; Ref., Reference. Total confirmed cases per million population (Confirmed cases) and total deaths per million population (Deaths) for COVID-19 are calculated.
