# Supplementary material for: Association between HLA gene polymorphisms and mortality of COVID‐19: An in silico analysis
Source: Immun Inflamm Dis. 2020 Oct 13;8(4):684–94. doi: 10.1002/iid3.358 (PMC7654404; doi:10.1002/iid3.358)
Supplement: Supplementary file 5 — Supporting information. [file IID3-8-684-s005.docx]

**Supplemental Material**

**Supplemental Figure**

**Supplementary Figure S1.** Global HLA allele distribution for HLA-A*02:01, -A*11:01 and -A*24:02. **World map depicting distribution of** HLA-A*02:01, -A*11:01, and -A*24:02 alleles **was created with mapchart.net.**

# Supplementary Figure S2. Number of predicted SARS-CoV2-derived T cell antigens. Comparisons of the numbers of predicted SARS-CoV2-derived T cell antigens between HLA-A*02:01, HLA-A*11:01 or HLA-A*24:02 are shown. For each HLA class I allele analyzed, we selected the top 0.5% (upper panel), 0.5% < percentile rank ≤ 1% (middle panel) and 1% < percentile rank ≤ 2% (lower panel) scoring peptides in the SARS-CoV-2 sequence, as ranked based on prediction. The SARS-CoV-2 protein sequences were run against HLA alleles using the NetMHCpan EL 4.0 algorithm available at the IEDB (<http://tools.iedb.org/mhci/>) and a size range of 8-11mers.

**Supplemental Tables**

**Supplementary Table S1.** Results of univariate regression analysis as of April 24^th^, 2020.

**Supplementary Table S2.** Results of analysis of covariance (ANCOVA) as of April 24^th^, 2020.
